# Evaluation of a midwifery network to guarantee outpatient postpartum care: a mixed methods study

**DOI:** 10.1186/s12913-020-05359-3

**Published:** 2020-06-22

**Authors:** Susanne Grylka-Baeschlin, Carolina Iglesias, Rebekka Erdin, Jessica Pehlke-Milde

**Affiliations:** 1 Research Unit for Midwifery Science, ZHAW Zurich University of Applied Sciences, Technikumstr. 81, CH-8401 Winterthur, Switzerland; 2 Familystart Zurich, Thurgauerstrasse 39, CH-8050 Zurich, Switzerland

**Keywords:** Postpartum care, Midwifery, Network, Psychosocially disadvantaged families

## Abstract

**Background:**

The necessity of outpatient postpartum care has increased due to shorter hospital stays. In a health care system, where postpartum care after hospital discharge must be arranged by families themselves, this can be challenging for those experiencing psychosocial disadvantages. Therefore, we compared characteristics of users of a midwifery network which referred women to outpatient postpartum care providers with those of women organising care themselves. Additionally, we investigated benefits of the network for women and health professionals.

**Methods:**

Evaluation of the services of a midwifery network in Switzerland. We combined quantitative secondary analysis of routine data of independent midwives with qualitative telephone interviews with users and a focus group with midwives and nurses. Descriptive statistics and logistic regression modelling were done using Stata 13. Content analysis was applied for qualitative data.

**Results:**

Users of the network were more likely to be: primiparas (OR 1.52, 95% CI [1.31–1.75, *p* < 0.001]); of foreign nationality (OR 2.36, 95% CI [2.04–2.73], *p* < 0.001); without professional education (OR 1.89, 95% CI [1.56–2.29] p < 0.001); unemployed (OR 1.28, 95% CI [1.09–1.51], *p* = 0.002) and have given birth by caesarean section (OR 1.38, 95% CI [1.20–1.59], *p* < 0.001) compared to women organising care themselves. Furthermore, users had cumulative risk factors for vulnerable transition into parenthood more often (≥ three risk factors: 4.2% vs. 1.5%, *p* < 0.001). Women appreciate the services provided. The collaboration within the network facilitated work scheduling and the better use of resources for health professionals.

**Conclusions:**

The network enabled midwives and nurses to reach families who might have struggled to organise postpartum care themselves. It also facilitated the work organisation of health professionals. Networks therefore provide benefits for families and health professionals.

## Background

In a health care system where out of hospital postpartum care has to be arranged by families themselves, this can be challenging for those experiencing psychosocial disadvantages. The first days after childbirth are a critical phase in the lives of mothers and their children and young families need support to overcome fears related to the transition into parenthood [1–4]. A safe early discharge from hospital requires access to guaranteed postpartum care at home in order to avoid readmission to hospital [5, 6]. In some health care systems in Central Europe, postpartum care after hospital discharge has to be organised by the families themselves [4, 7]. However, not all families have the necessary the knowledge and some of them need help. Mattern et al. [7] found in a German study that organising a midwife for postpartum care might be very stressful for women, especially if many phone calls are necessary to achieve success. Several studies have shown that psychosocially and economically disadvantaged families have limited access to postpartum care and use the provided services less frequently [8, 9]. Language barriers complicate the possibilities of organising care considerably [10, 11]. These families might need support for finding access to care.

Midwifery networks that organise and guarantee outpatient postpartum care facilitate access and increase the use of the services [12]. If outpatient postpartum care consists of home visits after hospital discharge, this provides a great opportunity to gain deep insight into the domestic situation [13–15]. The early support of disadvantaged families requires a well-functioning network involving various professionals such as employees of youth and social services, psychologists, physicians and others [16]. Midwives can assume an important role in such networks by identifying psychosocially disadvantaged families soon after birth and giving them follow up options such as early child support [17]. If psychosocially disadvantaged families find access to out of hospital postpartum care, a previous study showed that care was more extensive, because these families often need more support for the transition into parenthood [18]. Although more extended postpartum care generated additional costs, the overall health insurance expenditure during the first month after birth for mothers and their children remained stable after implementing a midwifery network, because the health care costs for the infants declined [12]. This means that costs for postpartum care are a good investment, because they prevent health problems and additional costs. Working in networks might also facilitate work scheduling for health professionals and enable a better use of their resources because the workload of postpartum care is irregular due to phases with high and low birth rates [19].

The length of hospital stay after childbirth declined in Switzerland from 5.7 days in 2001 to 4.4 days in 2015 (Federal statistical Office, Medical statistics of hospitals, unpublished data). As observed in other European countries, the implementation of Diagnosis Related Groups (DRG) for invoicing health care costs led to increased pressure on hospitals to discharge patients earlier [20]. The reason for this is that case based flat rates are invoiced to health insurances instead of the effective costs. In Switzerland, out of hospital postpartum care consisting of home visits by midwives is provided until the 56th day after birth and is regulated by the Health Care Insurance Act [21]. At the same time as the decline of the length of postpartum hospital stay, the use of out of hospital postpartum care provided by self-employed midwives and, to a lesser extent, also self-employed nurses with work experience in postpartum care, increased [22]. The majority of families in Switzerland make use of postpartum care after hospital discharge [22, 23]. However, postpartum care following hospital discharge has to be organised by the families themselves. Women find contact details for midwives and nurses in the internet but often need to call several health professionals to be successful. In some Swiss regions and during holiday periods, women have difficulties finding a midwife. The observed shift toward out of hospital postpartum care therefore emphasises the need for well-organised care providers. In different Swiss areas, midwifery networks assume the organisation of guaranteed out of hospital postpartum care. One of them is called “Familystart Zurich” and provides its services in the Canton of Zurich [24]. Women using these services register online themselves or are registered by the hospital and are mediated in an easy and uncomplicated way to postpartum care providers. Antenatal care providers and hospitals promote these services.

Hitherto, no study has investigated if midwifery networks reach families who would struggle to find access to out of hospital postpartum care without this support. Furthermore, no previous study has researched women’s and midwives’ views of these services.

## Methods

### Aim of the study

The aim of this study was to assess the benefits of guaranteed postpartum care through a midwifery network for families, especially socially disadvantaged ones, and health professionals. We investigated: a) how users of the services of the network differed from women organising their postpartum care themselves, b) women’s satisfaction and benefits and c) midwives’ and nurses’ satisfaction and benefits due to the services of the network.

### Study design and participants

We conducted a mixed method study design combining quantitative secondary analysis of routine data of Swiss independent midwives with telephone interviews with women who used the services of the midwifery network “Familystart Zurich” and focus group discussion with members of the network. Data of postpartum women living in the Canton of Zurich and giving birth in 2016 (*n* = 13,241) who either used the services of the network (*n* = 1544) to organise out of hospital postpartum care or organised it themselves (*n* = 11,697), were included in the secondary analysis. Women who used the services from the midwifery network with sufficient oral German language knowledge were eligible for the telephone interviews. Out of a total of 3108 users in 2016, 15 women were recruited evenly over a period of a year applying random selection stratified by parity, mode of birth, antenatal and postnatal registration as well as Swiss and foreign nationality. Midwives and nurses working in the network (*n* = 130) were invited to participate in the focus group discussion during the annual conference or were approached directly. Three midwives and two nurses consented to participate in the focus group discussion.

### Ethics

The Swiss Federation of Midwives gave written permission to use anonymised routine date for the purpose of the current study. Interview participants received oral and written study information and provided voluntarily written consent with the right to withdraw at any time. The Ethics Committee of the Canton of Zurich in Switzerland approved both, the quantitative (secondary analysis of routine data) and the qualitative part (telephone interviews, focus group discussion) of our study (BASEC-Nr. Req-2017-00192).

### Development of the interview guides

The interview guides were developed based on literature researches (supplementary files S1, S2). Studies investigating patient satisfaction with the organisation of service providers revealed themes which were addressed during the telephone interviews with the users of the services of the network: access to the offers, waiting time, friendliness of contact persons and fees [24, 25]. The interview guide for the semi-structured focus group discussion was adapted from the telephone interviews with the women in order to collect information about similar topics from different angles to enable triangulation. Additionally, job and occupational satisfaction of the midwives and nurses was addressed including the four themes of the subscales of the midwifery specific instrument from Turnbull et al. [26] professional satisfaction, professional support, client interaction and professional development. These themes were used as subjects of discussion.

### Data collection

Since 2005, routine data of independent midwives has been collected by the Swiss Federation of Midwives for quality insurance. To achieve full census, anonymised data is collected after oral information and consent (72,017 women in 2016). Data is analysed and results are published annually [22]. The variables “residential Canton of the mother” and “infant’s birth date” allowed the identification of women living in the canton of Zurich and giving birth in 2016 (*n* = 13,241). In order to identify the users of the network “Familystart Zurich”, 147 midwives were asked to provide the ID-number of Familystart Zurich users who they cared for and who gave birth in 2016 in the routine data set of independent midwives. A total of 110 midwives responded to this request (74.8%), allowing the identification of 1791 women. This number was lower than the registered 3108 users of the network in 2016, but not all of them gave birth in 2016 (registration at the end of 2016 and infant birth date in the beginning of 2017) and data of women who were cared for by nurses were not included in the routine data of independent midwives. Out of *n* = 1791 women, *n* = 1544 lived in the Canton of Zurich.

Qualitative data was collected by telephone and focus group interviews. The interviews were audio recorded and transcribed verbatim.

### Data processing and analysis

From the routine data of independent midwives from the year 2016, women living in the canton of Zurich were extracted. Cases identified by members of the network as being “users of the network” were coded 1 and all the other cases were coded 0 for “women organising postpartum care themselves”. Users of the network were encouraged by antenatal care providers and hospitals to register for these services. Women organising care themselves searched the Internet for personal details of midwives and contacted them themselves. Risk factors for a vulnerable transition into parenthood with associated risks for impaired child development and for child abuse such as teenage mother, single mother, no vocational education, jobless, poverty, migration, drug abuse, domestic violence, multiples, preterm birth or child with congenital malformation were investigated as single and cumulative factors [27, 28]. Descriptive statistics were computed according to the type of variables and study groups were compared using chi squared tests for categorical variables and Mann-Whitney-U-test for metric variables. Logistic regression modelling was carried out with the outcome variable “user of the network” yes or no. Potential predictors were variables which were significantly associated with the outcome variable, namely the variables “age”, “parity”, “nationality”, “living in partnership”, “professional education”, “employment” and “mode of birth”. Backward elimination was done for variables which did not remain significantly associated with the outcome variable in the model and age was kept in the model independently of the significance of the association. The logistic regression model was computed with and without the variable “parity”, because parity showed 28.8% of missing values due to technical problems of the data collection tool [22]. Due to the importance of the variable and the statistically significant association with the outcome variable, sensitivity analysis with and without the variable “parity” were conducted and results including “parity” are presented. Quantitative analyses were done using Stata 13 (StataCorp, Tx, USA).

Swiss German interviews were transcribed in German as is common practice, because Swiss German is not a written language. Typical Swiss German words with no clear equivalent in German were maintained. The transcribed texts were analysed using content analysis following the method of Mayring 2015 [29] and using the software Atlas.ti, version 8. Citations were linguistically polished in order to enhance comprehensibility and readability and translated into English if used for this publication. Translations were checked by two bilingual persons, one midwifery researcher with German mother tongue and one language instructor with English mother tongue.

## Results

Out of 3108 women referred in 2016 by the midwifery network Familystart Zurich to out of hospital postpartum care, 1544 were identified in the routine data of Swiss independent midwives as living in the canton of Zurich. Users of the network were compared with 11,697 women organising their postpartum care themselves and living in the same canton.

### Sociodemographic and perinatal characteristics

**Fig. 1:**
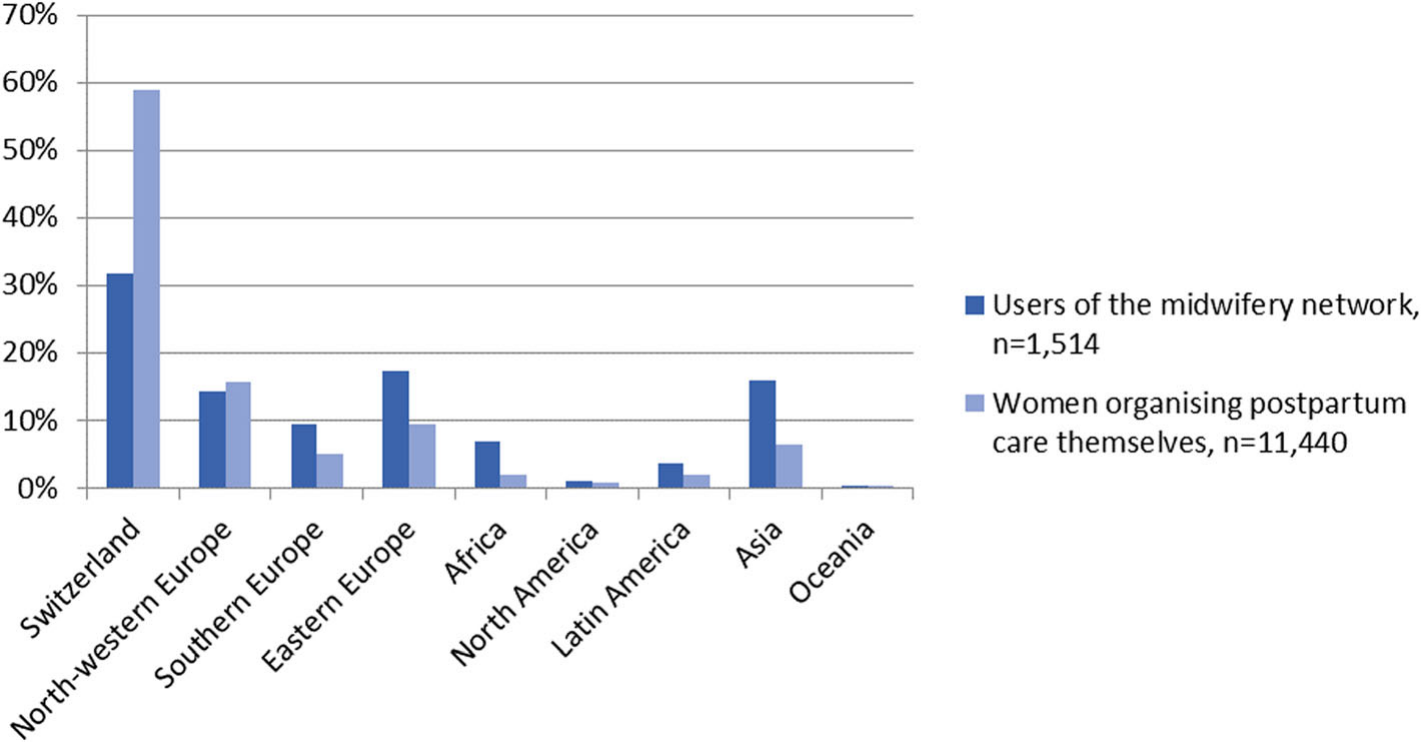
Nationalities grouped by geographic regions in users of the midwifery network compared to women, who organised postpartum care themselves

The comparison with women who organised midwifery postpartum care themselves showed that users of the network were slightly younger (median age: 32 years vs 33 years, *p* < 0.001) and less often of Swiss nationality (31.6% vs 58.7%, p < 0.001, Table 1). Regarding foreign nationalities grouped by geographic regions and continents, users of the midwifery network came slightly but not significantly less often from North-western Europe (14.2% vs 15.7%, *p* = 0.135), but significantly more often from Southern Europe (9.3% vs 5.0%, *p* < 0.001), Eastern Europe (17.4% vs 9.5%, p < 0.001), Africa (6.9% vs 2.0%, p < 0.001), Latin America (3.6% vs 1.9%, p < 0.001) and Asia (15.9% vs 6.3%, p < 0.001, Fig. 1). There was no significant difference in women coming from North America (1.0% vs 0.8%, *p* = 0.341) and Oceania (0.3% vs 0.2%, *p* = 0.502) between study groups. Users of the services of the network had no vocational education significantly more often (25.7% vs 9.3%, *p* < 0.001) and were less often employed before giving birth (62.4% vs 75.5%, *p* < 0.001). However, if employed, they worked full time significantly more often (48.3% vs 36.8%, *p* < 0.001).

**Table 1:** Sociodemographic characteristics of users of the midwifery network compared to women, who organised postpartum care themselves

Characteristics	Whole study population ***n*** = 13,241	Users of the midwifery network^a^ ***n*** = 1544	Women organising postpartum care themselves^a^ ***n*** = 11,697	***p***-value
***Sociodemographic characteristics***				
**Age** in years, md (min-max)	33 (16–59)	32 (17–56)	33 (16–59)	< 0.001
**Swiss nationality**, n (%)	7202 (55.6)	479 (31.6)	6723 (58.7)	< 0.001
**Living in partnership**, (n (%)	12,682 (98.4)	1445 (97.0)	11,237 (98.5)	< 0.001
**Highest vocational education**				< 0.001
No voc. Education, n (%)	1408 (11.2)	374 (25.7)	1034 (9.3)	
Apprenticeship/high school education, n (%)	5793 (46.2)	587 (40.3	5206 (47.0)	
Higher education/university, n (%)	5333 (42.6)	496 (34.0)	4837 (43.7)	
**Employed**, n (%)	9350 (74.0)	927 (62.4)	8423 (75.5)	< 0.001
**Full time employed**^b^, n (%)	3243 (37.9)	406 (48.3)	2837 (36.8)	< 0.001
***Obstetric and perinatal characteristic***				
**Parity**^c^				< 0.001
First child, n (%)	4741 (50.3)	682 (57.0)	4059 (49.3)	
Second child, n (%)	3466 (36.8)	350 (29.2)	3116 (37.9)	
Third child and more, n (%)	1218 (12.9)	165 (13.8)	1053 (12.8)	
**Mode of birth**				< 0.001
Spontanous vaginal, n (%)	6984 (55.2)	748 (48.6)	6236 (56.2)	
Instrumental vaginal, n (%)	1208 (9.6)	138 (9.0)	1070 (9.6)	
Caesarean section, n (%)	4452 (35.2)	652 (42.4)	3800 (34.2)	

^a^ All living in the canton of Zurich

^b^ Before giving birth, out of women, who were employed, *n* = 8556

^c^ 28.8% missing values

Additionally, women using the services of the midwifery network were significantly more often first-time mothers (57.0% vs 49.3%, p < 0.001), had their second child less often (29.2% vs 37.9%, p < 0.001) but similarly often their third child or more (13.8% vs 12.8%, *p* = 0.342) compared to women organizing their postpartum care themselves. Furthermore, users of the network gave birth spontaneously significantly less often (48.6% vs 56.2%, *p* < 0.001), had an instrumental vaginal birth similarly often (9.05% vs 9.6%, *p* = 0.408) but a caesarean section more often (42.4% vs 34.2%, p < 0.001).

### Risk factors

Compared to women organizing out of hospital postpartum care themselves, users of the midwifery network had the following social and medical risk factors for vulnerable transition into parenthood significantly more often: single mother (3.0% vs 1.5%, *p* < 0.001), no vocational education (25.7% vs 9.3%, p < 0.001), jobless (5.6% vs 3.0%, p < 0.001), poverty (1.7% vs 0.3%, p < 0.001), migration (7.1% vs 3.3%, p < 0.001), multiples (2.9% vs 1.8%, *p* = 0.007), preterm birth (7.7% vs 5.3%, p < 0.001) and child with congenital malformation (1.3% vs 0.8%, *p* = 0.045, Table 2). There was no significant difference regarding young mothers < 18 years, multiparous women < 20 years, drug abuse, domestic violence and suspected child abuse as well as depression. Users of the network also had no risk factor less often (57.4% vs 77.8%, *p* < 0.001) but more often one (29.2% vs 16.5%, p < 0.001), two (9.3% vs 4.3%, p < 0.001) and three or more risk factors (4.2% vs 1.5%, p < 0.001) than women organising postpartum care themselves.

**Table 2:** Risk factors in users of the midwifery network compared to women, who organised postpartum care themselves

Risk factor/number of risk factors	Whole study population n = 13,241	Users of the midwifery network^a^ n = 1544	Women organising postpartum care themselves^a^ n = 11,697	***p***-value
***Risk factor***				
**Young mother < 18 years**, n (%)	37 (0.3)	4 (0.3)	33 (0.3)	0.872
**Multiparous women < 20 years**, n (%)	5 (0.1)	0	5 (0.1)	1.000^b^
**Single mother**, n (%)	213 (1.7)	44 (3.0)	169 (1.5)	< 0.001
**No vocational education**, n (%)	1408 (11.2)	374 (25.7)	1′034 (9.3)	< 0.001
**Jobless**, n (%)	375 (3.4)	76 (5.6)	299 (3.0)	< 0.001
**Poverty**, n (%)	46 (0.5)	18 (1.7)	28 (0.3)	< 0.001
**Migration**, n (%)	353 (3.8)	78 (7.1)	275 (3.3)	< 0.001
**Drug abuse**, n (%)	3 (0.0)	1 (0.1)	2 (0.0)	0.310^b^
**Domestic violence or suspected child abuse**, n (%)	20 (0.2)	3 (0.3)	17 (0.2)	0.501^b^
**Depression**, n (%)	149 (1.6)	23 (2.1)	126 (1.5)	0.147
**Multiples**, n (%)	259 (2.0)	44 (2.9)	215 (1.8)	0.007
**Preterm birth**, n (%)	735 (5.6)	119 (7.7)	616 (5.3)	< 0.001
**Child with congenital malformation**, n (%)	113 (0.9)	20 (1.3)	93 (0.8)	0.045
***Number of risk factors***				
**No risk factor**, n (%)	4692 (75.2)	456 (57.4)	4236 (77.8)	< 0.001
**One risk factor**, n (%)	1129 (18.1)	232 (29.2)	897 (16.5)	< 0.001
**Two risk factors**, n (%)	307 (4.9)	74 (9.3)	233 (4.3)	< 0.001
**Three or more risk factors**, n (%)	112 (1.8)	33 (4.2)	79 (1.5)	< 0.001

^a^ All living in the canton of Zurich

^b^ Fisher’s exact test

### Predictors for the use of the services of the midwifery network

Multivariable analyses with the outcome variable “user of the network” yes or no were computed with and without the variable “parity” because of missing values for this parameter. Results of the logistic regression including the variable parity are shown in Table 3. Women with foreign nationality (OR 2.36, 95% CI [2.04–2.73], *p* < 0.001), without vocational education (OR 1.89, 95% CI [1.56–2.29], p < 0.001), who were not employed (OR 1.28, 95% CI [1.09–1.51], *p* = 0.002) or had given birth by caesarean section (OR 1.38, 95% CI [1.20–1.59], *p* < 0.001) were more likely to use the services of the midwifery network to gain access to out of hospital postpartum care. More highly educated women (OR 0.85, 95% CI [0.72–0.99], *p* = 0.003), having the second child (OR 0.62, 95% CI [0.53–0.73], p < 0.001) or their third child and more (OR 0.79, 95% CI [0.64–0.98], *p* = 0.035) were less likely to be users of the midwifery network.

**Table 3:** Logistic regression: Predictors for the use of the services of the midwifery network, *n* = 8384

Predictor	Odds Ratio	95% confidence interval	***p***-value
**Age** in years	0.99	0.98–1.00	0.143
**Nationality**			
Foreign nationality (Reference category Swiss)	2.36	2.04–2.73	< 0.001
**Highest vocational education**			
No vocational education	1.89	1.56–2.29	< 0.001
Higher education/university (Reference category apprenticeship/ high school education)	0.85	0.72–0.99	0.033
**Employed**			
Not employed	1.28	1.09–1.51	0.002
**Parity**			
Second child	0.62	0.53–0.73	< 0.001
Third child or more (Reference category first child)	0.79	0.64–0.98	0.035
**Mode of birth**			
Instrumental vaginal birth	0.97	0.77–1.24	0.832
Caesarean section (Reference category spontaneous vaginal birth)	1.38	1.20–1.59	< 0.001

### Women’s satisfaction and benefits

Participants of the 15 telephone interviews were on average 35.9 years (range 27–57 years) old. Two-thirds of them spoke German without any accent and were from Switzerland, Germany or Austria. One third of the interviewed users of the midwifery network spoke broken to fluent German with an accent and were from Kenya, Angola, Slovakia, Belarus and Poland. Despite addressing equal numbers of women registering before and after having given birth to ask if they would like to be interviewed, 86.7% (*n* = 13) of the participants registered during pregnancy. Nearly half of the women mentioned stressful situations: Migration, multiples miscarriages and preterm birth in history or age over 50 years. Additionally, the majority of women (*n* = 12) noted peculiarities or pathologies during pregnancy, birth or the postpartum period such as bleeding during pregnancy, premature labour, gestational diabetes, pelvic floor problems, twins, foetal tachycardia, unplanned repeat caesarean section, emergency caesarean section, vaginal birth after caesarean section, traumatic birth, referral to neonatal intensive unit or increased weight loss of the new-born.

The statements of the women on their satisfaction with the services of the network and on the benefits of its use were grouped into three themes: “Organising midwifery care”, “The services of the midwifery network” and “Postpartum care”.

#### Organising midwifery care

Not all interviewed women had information about the possibilities of out of hospital postpartum care and how to organise it.

*“(…) I waited quite a long time, because I did not really know how to find a midwife (…)”*

The search for a midwife proceeded very differently. Some users of the midwifery network were registered by the hospital without understanding how the registration and the organisation of midwifery care took place. Women registering themselves for the services of the midwifery network reported very different experiences trying to organise out of hospital postpartum care beforehand. Some of them were very frustrated while trying to organise it themselves or experienced difficulties organising postpartum care for a previous child.

*“… I looked on the special websites with addresses of midwives and (…) it turned out to be very difficult; so sometimes (…) I did not even receive a response and sometimes “I’m sorry, I’m fully booked, I have no capacity” (…)” “(…) I asked ten midwives, if they would have time and all of them declined (…)”.*

It was an issue for some women that they could not choose the midwife themselves or influence the allocation when using the services of the midwifery network. Thus, the midwife remained anonymous until postpartum care started.

*“(…) if I had had the possibility to choose, I would have looked for a midwife who I could at least have seen a picture of or perhaps have spoken to on the telephone”.*

However, other women did not have concrete ideas or expectations about the midwife and were not challenged with the impersonal organisation of care.

*“I did not look for (…) a particular person because I did not have any previous experience (…). But it was important for me to have a midwife with professional experience and this was actually my only criterion.”*

#### The services of the midwifery network

Some participants of the telephone interviews were registered with the midwifery network by the hospital staff. These women did not have any difficulty accessing the services and some of them did not realise how the organisation of the out of hospital postpartum care proceeded.

*“It was good, even perfect (…) she organised me (…) the midwife, and it was ok (…). I did not want to stay in hospital long because I had a caesarean section.”*

Service-users who tried to organise out of hospital postpartum care themselves highly appreciated the services and were very grateful about the easy and straightforward support which saved time, worries and stress.

*“(…) I can really express my thanks again for the straightforward and quick help and also that I had such a lovely midwife (…). It is really amazing that this offer exists (…). It was a great help then and decreased my worries and saved me time and for me, it was really great. I could actually not believe that it was so straightforward and easy and free. And my husband thought (…) yes, why did you call so many (midwives) yourself.”*

The contact with the midwifery network mostly took place by e-mail. Women who had contact with the office of the network reported that the communication was very friendly and professional.

*“(…) I think it was very professional and kind and that/that was enough for me, so, it was just good like this.”*

#### Postpartum care

Satisfaction with postpartum care was very high for the participants of the telephone interviews and for many women, the midwife evolved into an important reference person.

*“(…) she (the midwife) was very (…) empathic and friendly. And she was kind of a lifesaver for me. She was (uh) (..) I have to cry when I think about her, because it was (uh) a really difficult start for me”.*

Women with migration background recognised the midwife as a cultural mediator.

*“(…) and for me, it is good, because I profited from two experiences, from my mother’s and from the midwife’s (…) and I am very happy about this.”*

Some women also appreciated it if the midwife respected their individual boundaries. However, women needed confirmation that they handled the new situation well.

*“I have very positive memories. I was very happy that she (the midwife) was not intrusive (…) because, yes, I was a little bit afraid about people who know better. But she was (…) very pleasant. She saw that we were both good together (…). And she said that everything worked out very well (…) and yes (…) it was very pleasant.”*

An important point was the accessibility and reliability of the midwife. Women became nervous if it was difficult to reach the midwife or if she did not adhere to meeting times and was late.

*“… this is the only thing I have to say which was not so good. Because, after the baby’s birth, my husband sent a SMS and called the number which we received. And for two-three days, we did not get an answer.”*

### Midwives’ and nurses’ satisfaction and benefits

Three midwives and two nurses specialising in postpartum care participated in the focus group discussion. Their mean age was 52.4 years (43–57 years) and they had 23.3 years of professional experience (16–30 years). Participants had been self-employed for an average of 12 years (1–26 years) and in 2016, cared for 132 women (10–250 women) on average.

The statements of the midwives and nurses were grouped into four themes: “The membership in the network”, “Use of resources”, “Caring for users of the network” and “Job satisfaction”.

#### The membership in the network

The network operates a mobile application providing an overview of women who are looking for a midwife. Members of the network appreciated that this facilitated their work organisation.

*“I see a clear advantage that I get an overview of women (who are looking for a midwife) and that it states where they live, their due date and some additional information. This already helps me to (…) prepare my schedule.”*

However, the introduction of new technologies required some effort for the members.

*“This is a technical problem; how does this app really work. Why did they have to change this (…).”*

The midwifery network is also active in the interface management with the hospitals and the follow-up support offered to families. Members appreciated the contacts of the network with the hospitals and the follow-up offers but would appreciate if the network would operate as an ombudsman service for feedbacks to the hospital about women and their children who, according to midwives’ assessment, left the hospital in a poor state of health.

*“We created a new transfer form (for the follow-up offers) and we work very well with this.”*

*“(…) if it were possible to put the complaints (to the hospitals) to a neutral ombudsman service, without annoying people or having to go to different hospitals myself.”*

#### Use of resources

The beginning of care for women who register before giving birth may vary by several weeks around the due date because of the uncertainty about the birth date of the baby. This often leads to an imbalanced workload. Participants of the interview emphasised that women who register to the network at short notice enabled gaps in their workload to be filled.

*“(…) you can fill all your work gaps very well. So, you have almost no risks anymore. You even need to check that you do not work too much.”*

Being able to have an overview of women who are looking for a midwife provides the possibility to choose those close to the midwife’s home or to other patients and therefore to keep routes short.

*“I have almost no travel time anymore (to visit women). I walk a lot (…). Thus, you can save a lot of time.”*

#### Caring for users of the network

Members of the midwifery network observed that women using the services of the network differed from women organising postpartum care themselves. Users of the network often did not have knowledge about the Swiss maternity care system.

*“Hence, there are certainly more vulnerable families (…) because the others, who can find a midwife themselves, they realised that it would be good to have follow-up-care.”*

*“But even certain women, (…) who have a higher educational level (…) do not know it (that they should organise care themselves).”*

Midwives and nurses experience a sincere gratitude from users of the network but also a great responsibility when caring for vulnerable families. Home visits allow a deep insight into their family lives and enable for example observation of older siblings’ behaviour.

*“They [the women] are very grateful if you say “yes (…) I will come tomorrow, no problem and I will bring a breast pump” and then you know that they already feel a bit better (…). And because they are desperately looking for a midwife and sometimes when they leave the hospital, they do not know if somebody will come the next day (…) they are really grateful.”*

*“We have an insight into these families and see how parents care for the siblings. We can observe for example if a three-year-old (sibling of the baby) is sitting in front of the television all day.”*

Midwives were also confronted with language difficulties and would have appreciated the support of interpreters with cultural mediation skills.

#### Job satisfaction

The midwives and nurses were generally very satisfied with their work as self-employed midwives but had difficulties to distinguish between general job satisfaction and satisfaction due to their participation in the network. Services of the network such as enabling the possibility to choose women who lived near to each other or close to the midwife’s home and therefore, avoiding long journeys for the home visits seem to increase satisfaction.

*“… I’m very, very satisfied, I have to say (…). It is also very nice to travel by bike…. It is great to ride the streets with the e-bike…”.*

## Discussion

Our study showed the great value of organised and guaranteed postpartum care for socially disadvantaged families and midwives. The network was able to reach families who might have been challenged to organise postpartum care themselves in a health system, in which it is normal practice to do so. Additionally, women appreciated the services very much and confirmed the need of support for organising postpartum care. Being a member of the network enabled midwives and nurses to better organise their work and use their resources and job satisfaction of the professionals was high.

Users of the network differed from women organising out of hospital postpartum care themselves and were more frequently primiparae, of foreign nationality, had vocational training less often, were more frequently unemployed and gave birth more often by caesarean section. They also showed one or several risk factors for a vulnerable transition into parenthood leading to an unfavourable environment for child development more often. Our results could also be compared with unpublished cumulative data from 2013 to 2015 of the general population provided from the statistical office of the canton of Zurich. In the population of women of childbearing age, only 39.8% were of foreign nationality compared with 68.4% of the users of the network. The proportion of foreigners in the users of the network was even higher than the one in the largest hospital (University Hospital Zurich, personal communication, 2017) which had a contract with the network (68.4% vs 59.4%). The difference between users of the network and other women (68.4% vs 44.4%) was also higher than the general difference between city and canton of Zurich (39.8% versus 33.5%) (Statistical Office Canton of Zurich, Cumulative data 2013-2015, unpulished data). This was especially important because more than half of the users of the network lived in the city. Additionally, the high proportion of women originating from Southern and Eastern Europe as well as from Asia and Africa showed that users of the network often had cultural backgrounds which were fundamentally different from the Central European one in which they lived. Comparing the highest vocational education of the users of the network with the general population of women of childbearing age showed a similar picture. The proportion of woman without vocational education was 13.7% in the city of Zurich and 16.4% in the whole canton of Zurich (inclusively city) (Statistical Office Canton of Zurich, Cumulative data 2013-2015, unpulished data) compared to 25.7% in the users of the network. The proportion of caesarean section of users of the network (43.2%) was also higher than the average in the three contract hospitals (39.0%) (Familystart Zurich, caesarean section rate in contract hospitals 2016, personal communication 2017) and higher than in most city areas of Zurich (32.6–44.5%) and higher than the cantonal average (37.2%) [30].

Previous studies showed that psychosocial disadvantages and language barriers prevent women from having access to postpartum care [8–11]. Our study findings indicate that burdened families, who were more often of foreign nationality, with no vocational training and had more risk factors for impaired transition into parenthood, might have more difficulty finding access to care but offering them support, enables it. This is especially important in the vulnerable period after giving birth to promote a safe transition into parenthood [2–4]. Erdin et al. [18] found in a secondary analysis of routine data of Swiss independent midwives of the whole country that women with risk factors needed even more care and home visits. Our analyses showed that despite this additional need for support, burdened families who often do not have enough knowledge about the health care system, would have fallen through the cracks of maternity care if they could not have profited from the services of the network. This confirms the findings of Wilcox et al. [10] that socially and economically vulnerable families did not use the proposed postpartum care services. Moreover, our findings were also in line with the ones of a German study which found that women with psychosocial problems and limited literacy are overwhelmed by organising midwifery care themselves [8]. It seems therefore very important that access to midwifery networks which support families in organising care should be facilitated and be promoted by antenatal care providers and hospitals.

Many midwives in Switzerland work very independently and are not organised in networks [19]. They often bear the risk of imbalanced workloads and burdens of difficult care situations themselves. The statement of midwives and nurses in the current study showed that being a member of a network helps to schedule work. However, the participants of the focus group discussion mentioned possibilities for improvement regarding the support of the network, such as their wish to have an ombudsman service for difficult feedbacks to the hospitals. Further research is needed to investigate which kind of support is necessary to ease the burden of postpartum health care providers, especially if they care for psychosocially disadvantaged families.

Strengths of our study were the relatively large data set for the comparison of women using the services of the midwifery network with those organising postpartum care themselves. Nevertheless, it was not possible to identify all users in the routine data of Swiss independent midwives and for some variables, the proportion of missing data was high. Users of the network who were not identified but gave birth in 2016 were wrongly allocated to the study group including women who organised care themselves. This bias would only have weakened our results which indicates that the significant differences between study groups was robust despite this small bias. Missing data is a known problem in observational studies [31]. Due to technical problems during data collection, the proportion was especially high for the variable parity. The logistic regression could therefore not be performed with a complete dataset. However, we could not identify any pattern in missing data which was related to the distinction between study groups, which reduces the risk of biased results. Sample sizes for the telephone interviews with users of the network and for the focus group discussion were small and results might not be fully representable for the study population. The stratified selection of potential candidates for the telephone interviews aimed for a heterogeneous study sample. Nevertheless, women registering during pregnancy and those speaking German well participated more often. Interviewed women were therefore not all socially disadvantaged but nevertheless, provided valuable information about the benefits of the services of the midwifery network. A further limitation was that midwives and nurses participating in the focus group discussion might have been the most engaged ones with positive views. Furthermore, discussing job satisfaction in a focus group might have prevented some participants from communicating all their concerns. Additionally, the health professionals cared for varying numbers of women per year leading to very different working situations. The workload might have influenced their statements because a higher one might have been associated with increased stress but maybe also with increased work engagement.

## Conclusion

Our study showed that through the services of a midwifery network psychosocially disadvantaged families who need support for a good transition into parenthood could be reached. They might not have been able to organise postpartum care themselves and might have fallen through the safety net of the supply network without this support. Guaranteed postpartum care eases the burden on families and reduces stress. As a consequence, families appreciated the services of the midwifery network very much. The network also facilitated midwives and nurses to schedule their work and enabled a better use of their resources. Hence, our study showed on the one hand, that the responsibility to organise care should not be left to the families, because especially psychosocially challenged ones are overburdened with this task. Access to such networks should be facilitated and promoted by antenatal care providers and hospitals. On the other hand, the study also demonstrated that working together in networks has many advantages for the midwives. Midwifery networks have therefore benefits for families as well as for health professionals. Future studies should investigate midwives’ needs for support when caring for burdened families such as access to interpreter services.

## Supplementary information

**Additional file 1.**

**Additional file 2.**

## Data Availability

The datasets generated and/or analysed during the current study are not publicly available due to confidentiality of data but are available from the corresponding author on reasonable request.

## Abbreviations

CI: Confidence interval
DRG: Diagnosis related groups
OR: Odds ratio
vs: versus
