# Supplementary material for: Evaluation of a midwifery network to guarantee outpatient postpartum care: a mixed methods study
Source: BMC Health Serv Res. 2020 Jun 22;20:565. doi: 10.1186/s12913-020-05359-3 (PMC7310082; doi:10.1186/s12913-020-05359-3)
Supplement: Supplementary file 1 — Additional file 1. [file 12913_2020_5359_MOESM1_ESM.pdf]

Interview guide for telephone interviews with the users of the midwifery network

1. Start of the conversation

- Introduction
- Information about the evaluation of the midwifery network
- Aim of the interview
- Indication of the use of the audio recorder and the anonymisation and pseudonymisation of data
- Completion of questionnaire about personal data (including time of registration)

2. Access to the services of the midwifery network Familystart

- How did you organise outpatient postpartum care? What went well and what was challenging?
- How did you decide that you would need outpatient postpartum care? How did you register with the midwifery network (yourself or hospital)?
- How much time did you need to organise outpatient postpartum care?
- Did you complete the registration form for the network yourself? How user-friendly was it?
- For multiparas who experienced the organisation of outpatient postpartum care without the network for their previous child/children: How was the organisation of outpatient postpartum care without the support of the midwifery network?

3. Time between registration and allocation of a midwife

- How was the time between registration and the allocation of a midwife?
- How long did you have to wait until outpatient postpartum care was organised for you?

4. Kindness of contact person

- Did you have personal contact with the office of the midwifery network Familystart?
- According to the answers: how did you experience this contact?
- According to the answers: how kind and professional was the contact person?

5. Costs (if birth did not take place in a contract hospital)

- What did the costs for the services mean to you?
- Do you think, that the costs were reasonable? If not, why was is not appropriate?

6. Postpartum care

- How did you experience the postpartum care provided by your midwife or your nurse?

7. Opportunity for improvement

- In your view, are there any aspects of the services that could be improved?
