# Supplementary material for: Evaluation of a midwifery network to guarantee outpatient postpartum care: a mixed methods study
Source: BMC Health Serv Res. 2020 Jun 22;20:565. doi: 10.1186/s12913-020-05359-3 (PMC7310082; doi:10.1186/s12913-020-05359-3)
Supplement: Supplementary file 2 — Additional file 2. [file 12913_2020_5359_MOESM2_ESM.pdf]

Interview guide for focus group discussion with midwives and nurses of the network

1. Start of the conversation

- Introduction
- Information about the evaluation of the midwifery network
- Aim of the interview
- Indication of the use of the audio recorder and the anonymisation and pseudonymisation of data
- Completion of questionnaire about personal data (inclusively time of registration)

2. Services of the midwifery network Familystart

- What are the advantages and the challenges of the services of the midwifery network and your membership?
- What does it mean for you to be member of the network?

3. Contact with the midwifery network

- How did you experience the contact with the office and the board of the network?

4. Costs for membership and services

- What is your opinion about the costs, which you pay for the services and the membership of the network?

5. Care for women who organised postpartum care through the network

- How is the care provision for women who organised outpatient postpartum care through the midwifery network?
- Are there any differences between users of the network and women organizing care themselves?
- What resources and support do you receive when you are confronted with difficulties (e.g. interpretation service, interprofessional networks)?

6. Job satisfaction (based on the themes of the subscales of the midwifery specific instrument of Turnbull et al. 1995)

- How do you experience your professional situation (general question)?
- How satisfied are you with your work (professional satisfaction subscale)?
- How is the collaboration with the midwifery network and with other midwives (professional support subscale)?
- If you are working in a midwifery team, how do you experience it? (e.g. collaboration with less experienced midwives)
- How do you experience the relationship with the women/families, who were allocated by the network (client interaction subscale)?
- How do you experience the opportunity for professional development (professional development subscale)?
- If midwives are unsatisfied, is this due to the services of the midwifery network or their work situation as self-employed midwives?

7. Economic questions

- How did membership of the network have an impact on the use of your resources and the planning or your work?

- How would you evaluate the impact of your care in preventing possible readmission to hospital?

8. Opportunity for improvement

- Do you have any recommendations for improvement of the network?
